# A two-gene-based prognostic signature for pancreatic cancer

**DOI:** 10.18632/aging.103698

**Published:** 2020-09-23

**Authors:** Shuyi Zhou, Yuanliang Yan, Xi Chen, Shuangshuang Zeng, Jie Wei, Xiang Wang, Zhicheng Gong, Zhijie Xu

**Affiliations:** 1 The Hunan Institute of Pharmacy Practice and Clinical Research, Xiangya Hospital, Central South University, Changsha 410008, China; 2 Department of Hepatobiliary Surgery, Hunan Provincial People’s Hospital Xingsha Branch, People’s Hospital of Changsha County, Hunan Normal University, Changsha 410008, China; 3 Department of Pharmacy, Xiangya Hospital, Central South University, Changsha 410008, China; 4 National Clinical Research Center for Geriatric Disorders, Xiangya Hospital, Central South University, Changsha 410008, China; 5 Department of Pathology, Xiangya Hospital, Central South University, Changsha 410008, China

**Keywords:** pancreatic cancer, ANLN, HIST1H1C, overall survival, prognosis

## Abstract

The purpose of this study was to identify a vital gene signature that has prognostic value for pancreatic cancer based on gene expression datasets from the Cancer Genome Atlas and Gene Expression Omnibus. A total of 34 genes were obtained by the univariate analysis, which were significantly associated with the overall survival of PC patients. After further analysis, *Anillin (ANLN)* and *Histone H1c (HIST1H1C)* were identified and considered to be the most significant prognostic genes among the 34 genes. A prognostic model based on these two genes was constructed, and successfully distinguished pancreatic cancer survival into high-risk and low-risk groups in the training set and testing set. Subsequently, independent predictive factors, including the age, margin condition and risk score, were then employed to construct the nomogram model. The area under curve for the nomogram model was 0.826 at 0.5 years and 0.726 at 1 year, and the C-index of the nomogram model was 0.664 higher than the others variables alone. These findings have indicated that high expression of *ANLN* and *HIST1H1C* predicted poor outcomes for patients with pancreatic cancer. The nomogram model based on the expression of two genes could be valuable for the guidance of clinical treatment.

## INTRODUCTION

Pancreatic cancer (PC), the fourth leading cause of cancer-related death in the US, is estimated to cause 227,000 deaths per year worldwide. As the incidence rate of PC in developed countries continues to rise, it will be the second most fatal cancer in 2020 [1]. Although modern cancer chemotherapy and mature surgical techniques cause a modest incremental improvement in patient outcomes [2, 3], with the 5-year overall survival (OS) improving from 5% to 9% and the median OS improving to approximately 11 months compared with the historic benchmark of 5 to 6 months, its prognosis still remains extremely poor [4]. Thus, efforts are needed to develop new ways to treat this fatal disease.

In recent years, precision medicine has offered numerous valuable insights into PC treatment [5]. Individual therapy enables PC patients to have the largest gains with minimum risk. Much more radical treatments, such as combined regimens and extensive radical operation, are preferred for PC patients with high recurrence risks [6]. Therefore, it is extremely necessary to distinguish the high-risk group from all PC patients.

However, there is no sufficient predictive system to predict the outcomes of patients with PC. Traditional risk stratification systems, such as the American Joint Committee on Cancer (AJCC) staging system, have been considered relatively nondiscriminatory for predicting differences in survival among PC patients [7, 8]. With the use of next-generation sequencing and microarray technologies, many studies have found the importance of gene signatures in the initiation, progression and prognosis of human tumors [9–13]. The facilitating investigation of interactions between gene signatures and tumors has made it possible to use signatures to stratify patient risks.

In this study, we aimed to explore the differences in mRNA expression profiles between PC and the adjacent pancreas using The Cancer Genome Atlas (TCGA) and Gene Expression Omnibus (GEO) datasets. After important prognosis-related genes were identified, we established a two-gene prognostic model that included *Anillin (ANLN)* and *Histone H1c (HIST1H1)* and was applicable for guiding prognostic assessment and treatment decision-making during the early postoperative period.

## RESULTS

### Common differentially expressed genes between PC and normal tissues

**Figure 1:**
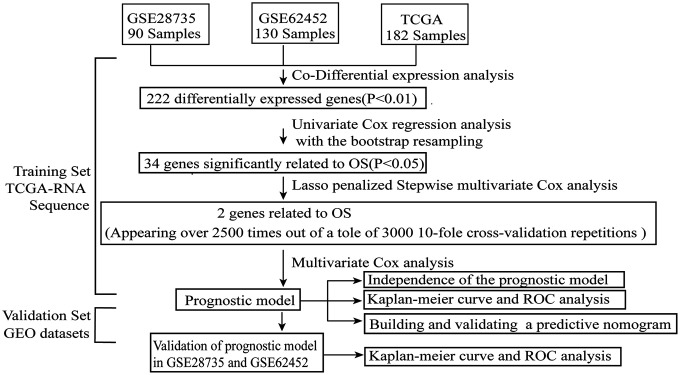
**The flowchart of the whole analysis process.**

**Figure 2:**
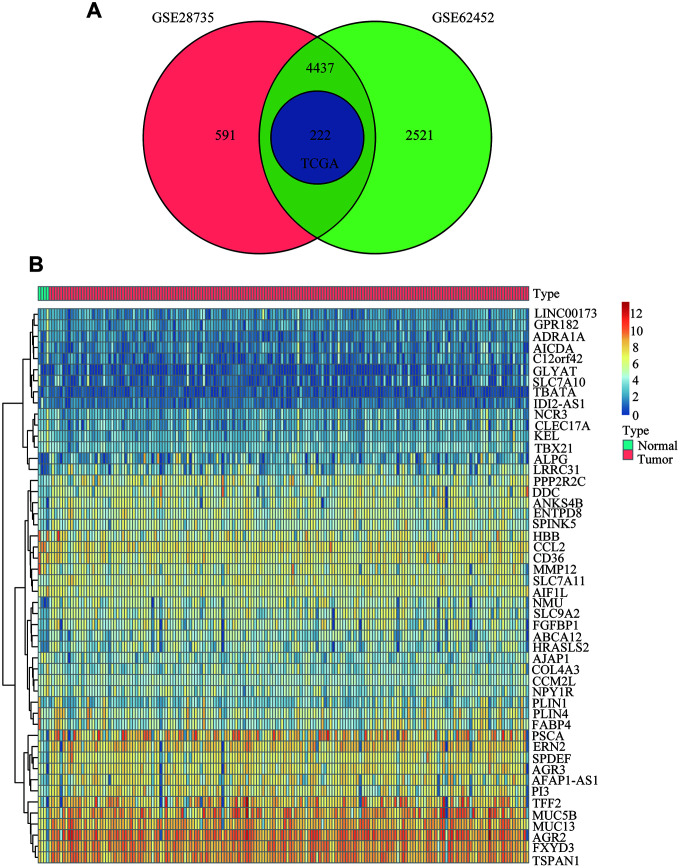
**Common differentially expressed genes between PC and normal tissues.** (**A**) Venn diagram showing the common DEGs in PC and adjacent normal tissues from the GSE28735, GSE62452 and TCGA datasets. (**B**) Heatmap analysis of the 222 DEGs, which contained the 50 highest expressed genes and the 50 the lowest expressed genes according to the log2FC between normal tissues and cancer tissues from the TCGA datasets.

As shown in the analysis process flowchart (Figure 1), after the genomic differential expression analysis of the 3 datasets (GSE28735, GSE62452 and TCGA), there were 222 differentially expressed genes (DEGs) in common (Supplementary Table 1). Using the criteria of P value < 0.01, the number of DEGs in GSE28735, GSE62452 and TCGA are 5250, 7180 and 222, respectively. A Venn diagram was applied to visualize the DEG relationships of the 3 datasets (Figure 2A). We also used a heatmap of the differentially expressed mRNAs to better differentiate normal tissues from PC (Figure 2B and Supplementary Tables 2, 3).

### Functional annotation of common DEGs

**Figure 3:**
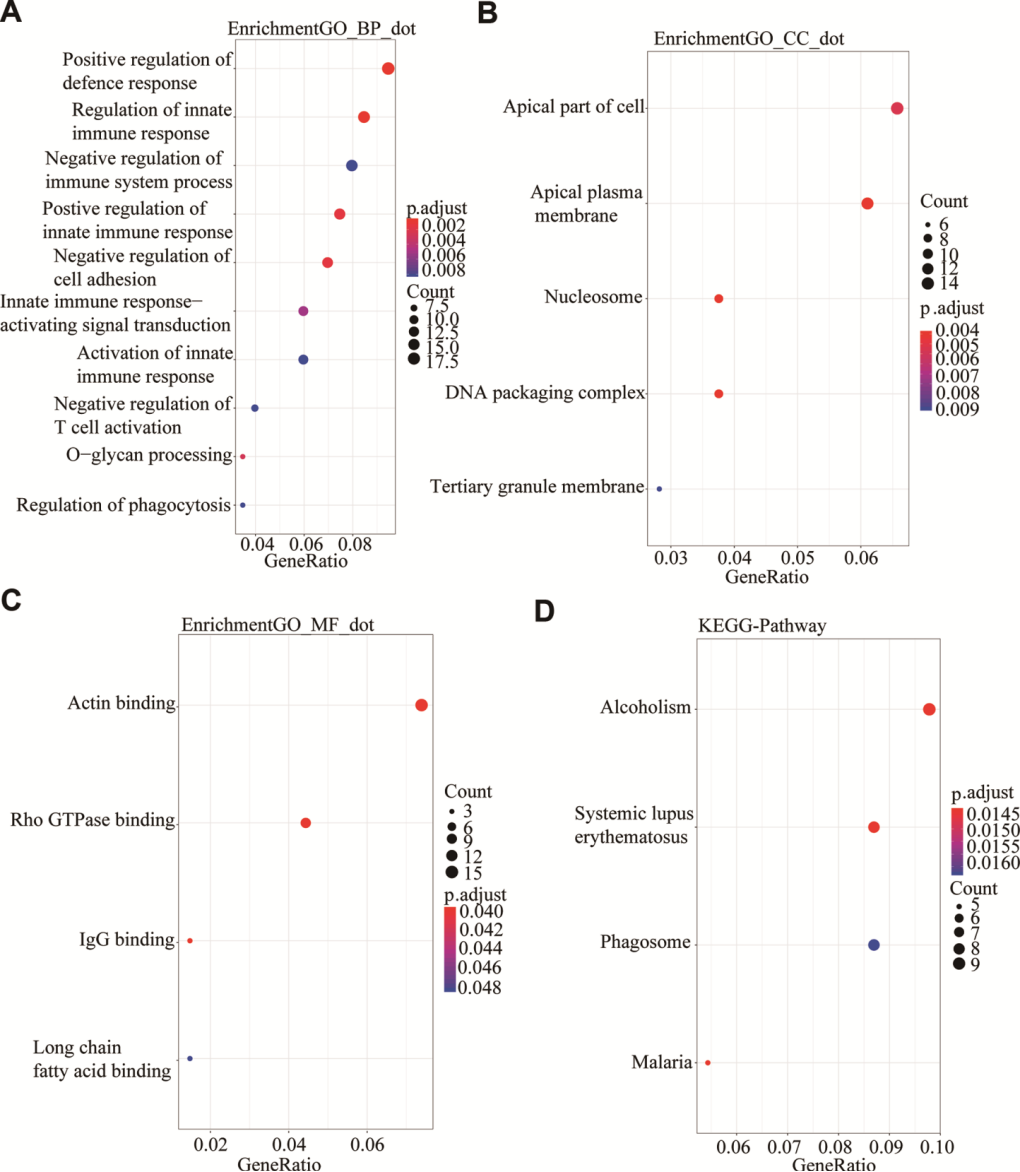
**GO and KEGG analysis using the R package “Clusterprofile” for the 222 common DGEs from the three databases.** P<0.05 was set as the threshold. (**A**) Biological process. (**B**) Cell component. (**C**) Molecular Function. (**D**) Kyoto Encyclopedia of Genes and Genomes.

To further describe the biological functions of the 222 common differentially expressed genes in detail, we performed functional annotation and enrichment analysis using the R package “ClusterProfile”. The biological process indicated that genes were enriched for positive regulation of defense response (Figure 3A). For cell component enrichment, the common differentially expressed genes were primarily enriched for apical part of cell (Figure 3B). The molecular function of the genes was enriched mainly for Alcoholism (Figure 3C). The KEGG pathway indicated that the DEGs were mainly enriched for actin binding (Figure 3D).

### Screening of the prognostic PC gene signature among DEGs

**Figure 4:**
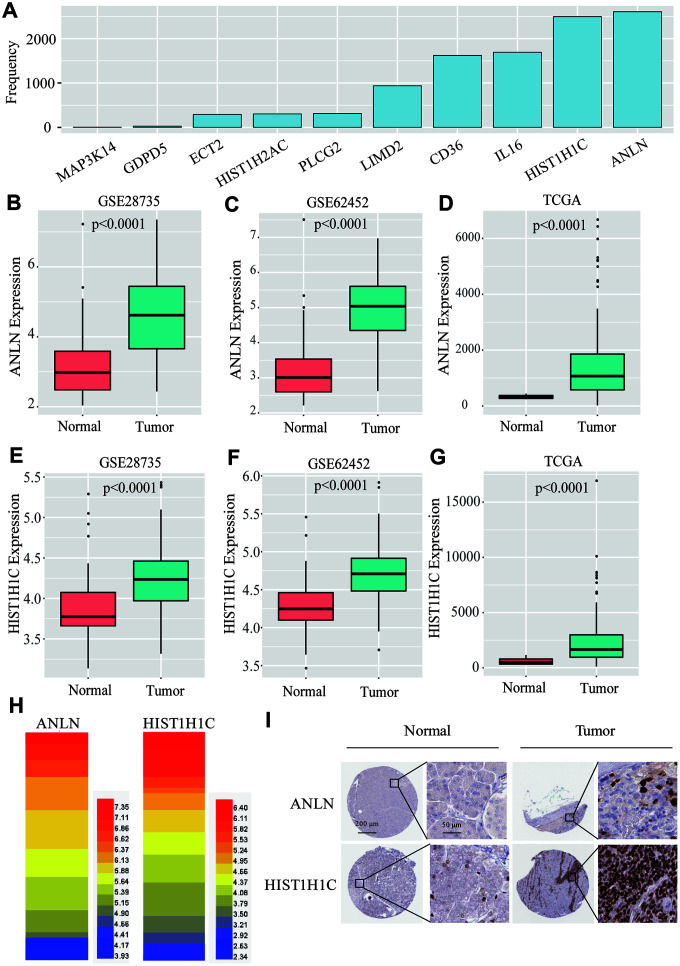
**Screening of the two-gene-based signatures in PC patients.** (**A**) After 3000 analyses, *ANLN* and *HIST1H1C* appeared more than 2500 times as the independent prognostic genes among 35 survival related genes. (**B**–**D**) The boxplot shows that ANLN were constantly high-expressed in GSE28735, GSE62452 and TCGA. (**E**–**G**) The boxplot shows that HIST1H1C were constantly high-expressed in GSE28735, GSE62452 and TCGA. (**H**) The heatmap of *ANLN* and *HIST1H1C* mRNA expression in PC cells from Cancer Cell Line Encyclopedia. (I) the Human Protein Atlas project shows representative immunohistochemical images of ANLN and HIST1H1C in PC tissues compared with surrounding normal tissues.

After a univariate Cox analysis with the bootstrap resampling, we found that 34 of the 222 common DEGs were significantly related to PC patient survival (P<0.05). Next, to further reduce the number of genes and overfitting, we subsampled 70% of patients from TCGA dataset (Supplementary Table 4) for analysis at one time and applied a Lasso-penalized regression with a 10-fold cross-validation 3000 times when performing the stepwise multivariate Cox analysis. Two genes, *ANLN* and *HIST1H1C*, appeared over 2500 times among the 3000 10-fold cross-validation repetitions (Figure 4A and Supplementary Table 5). Next, we used a box plot to illustrate that *ANLN* and *HIST1H1C* are constantly and significantly highly expressed in tumor tissues from all the datasets (GSE28735, GSE62452 and TCGA) (Figure 4B–4G and Supplementary Tables 6–8). Furthermore, the data from Cancer Cell Line Encyclopedia (https://portals.broadinstitute.org/ccle) were conducted as the heatmap displaying the elevated *ANLN* and *HIST1H1C* expression levels in several PC cells (Figure 4H and Supplementary Table 9). Next, we wanted to evaluate the protein expression levels of ANLN and HIST1H1C in PC patients. We analyzed the immunohistochemical data from the Human Protein Atlas (http://www.proteinatlas.org/) shown in Figure 4I, and found significantly elevated levels of ANLN and HIST1H1C in the tumor tissues. In addition, the pancancer analysis from GEPIA 2.0 (http://gepia2.cancer-pku.cn/#index) showed that upregulated *ANLN* and *HIST1H1C* transcripts are frequently observed in multiple cancer types, including PC (Supplementary Figure 1). Taken together, *ANLN* and *HIST1H1C* play potential oncogenic roles in most types of human cancers.

### The risk stratification and ROC curve indicate the good performance of the two-gene based signature

**Figure 5:**
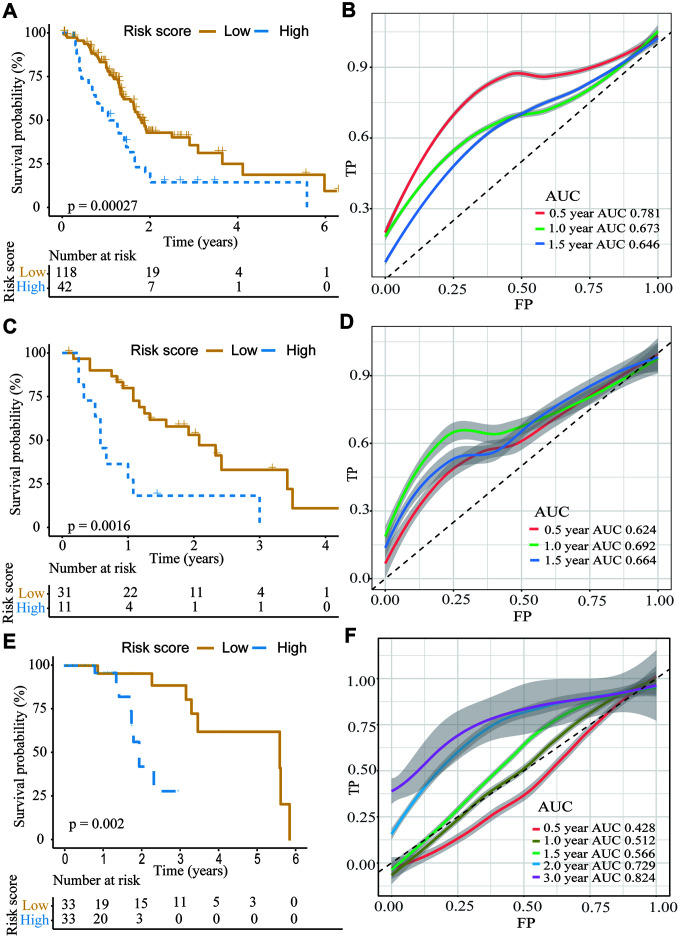
**The K-M plot showed a lower overall survival in the high risk group compared to the low risk group divided by the optimal cut-off point.** (**A**, **B**) K-M and time-dependent ROC curves for the prognostic model based on *ANLN* and *HIST1H1C* expression in the TCGA PC cohort. (**C**, **D**) K-M and time-dependent ROC curves for the prognostic model based on *ANLN* and *HIST1H1C* expression in the GSE28735. (**E**, **F**) K-M and time-dependent ROC curves for the prognostic model based on *ANLN* and *HIST1H1C* expression in the GSE62452.

Using the TCGA dataset, we generated a predictive model based on the expression of two genes, which was characterized by the linear combination of the expression levels of the two genes weighted by their relative coefficient in the multivariate Cox regression. We subsequently calculated the two-gene expression risk score and used X-tile diagrams to produce the optimal cut-off value for the risk score. According to the risk score cut-off point, 42 patients were classified into the high-risk group, and the remaining 118 patients were assigned to the low-risk group. As is shown in Figure 5A and Supplementary Table 10, the Kaplan-meier (K-M) OS curves of the two groups based on the two genes were significantly different (median OS, 1.81 years vs 1.08 years, P=0.00027). To assess the prognostic capacity of the two-gene signature, the area under curve (AUC) of a time-dependent ROC curve was calculated. The AUCs of the two-gene biomarker prognostic model were 0.781, 0.673 and 0.646 for the 0.5-, 1- and 1.5-year survival times, respectively (Figure 5B). To further evaluate the generality of the two-gene biomarker prognostic model, we verified the model with the GEO dataset (GES28735), which contains both mRNA expression and clinical survival data from 45 PC patients. Using the same data management as in the TCGA, we also calculated the two-gene risk score according to the expression levels in GES28735 and the coefficient of the multivariate Cox regression and found an optimal cut-off value for the risk score by means of the X-tile diagrams. A total of 42 PC patients in the GSE28735 dataset were classified into high-risk group (n=11) and low-risk group (n=31). Consistent with the results in the TCGA, and as is shown in Figure 5C and Supplementary Table 11, the K-M OS curves indicated that the OS of PC patients included in the GSE28735 data in the high-risk group was significantly lower than that in the low-risk group (median OS 0.58 years vs 2.08 years, P=0.0016). Moreover, the time-dependent ROC analyses for the survival prediction of the prognostic model obtained AUCs of 0.624 at 0.5 years, 0.692 at 1 year and 0.664 at 1.5 years (Figure 5D). In addition, we further verified the risk model with the GEO dataset (GSE62452), which contains both mRNA expression and clinical survival data from 69 PC patients. A total of 66 PC patients in the GSE62452 dataset were classified into high-risk group (n=33) and low-risk group (n=33). Consistent with the results in the TCGA and GSE28735, and as is shown in Figure 5E and Supplementary Table 12, the K-M OS curves indicated that the OS of PC patients in the high-risk group from GSE62452 was significantly lower than that in the low-risk group (median OS 1.26 years vs 2.09 years, P=0.002). Moreover, the time-dependent ROC analyses for the survival prediction of the prognostic model obtained AUCs of 0.729 at 2 year and 0.824 at 3 years (Figure 5F). Above all, we concluded that the two-gene signatures were able to predict the prognosis in PC patients.

### Building and validating a predictive nomogram

**Figure 6:**
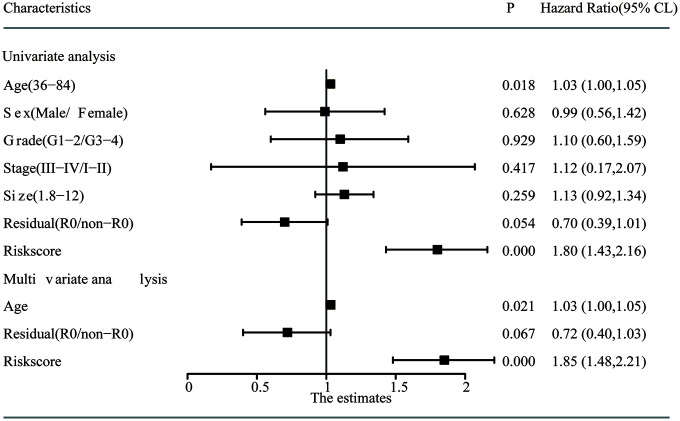
**Univariate and multivariate analysis of the risk score and clinicopathological characteristics with OS.** The residual (R0/non-R0) and risks core indicated the margin condition and prognostic model, respectively.

Univariate and multivariate Cox regression analyses were employed to detect the independent predictive ability of the two-gene-based prognostic model in the abovementioned TCGA PC cohort with detailed clinical information. Using the univariate Cox regression analysis, we found that the prognostic model and age had prognostic values, while the others variables did not significantly correlate with OS (Figure 6). Considering that the margin condition almost reached statistical significance (P=0.054) and might affect the prognosis of PC patients according to the clinical experience, we incorporated age, margin condition and prognostic model into the multivariate Cox regression analysis. As a result, both the age and prognostic model were independent prognostic factors, and the margin condition nearly reached statistical significance (Figure 6).

**Figure 7:**
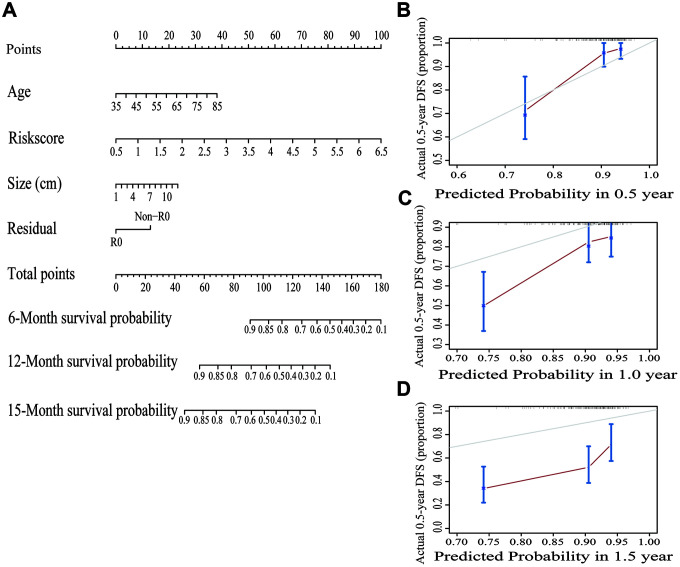
**Building and validating a predictive nomogram.** (**A**) A nomogram to predict survival probability at 6, 12 and 15 months after surgery. (**B**) Calibration curve for the nomogram when predicting 6 months of overall survival. (**C**) Calibration curve for the nomogram when predicting 12 months of overall survival. (**D**) Calibration curve for the nomogram when predicting 15 months of overall survival.

**Figure 8:**
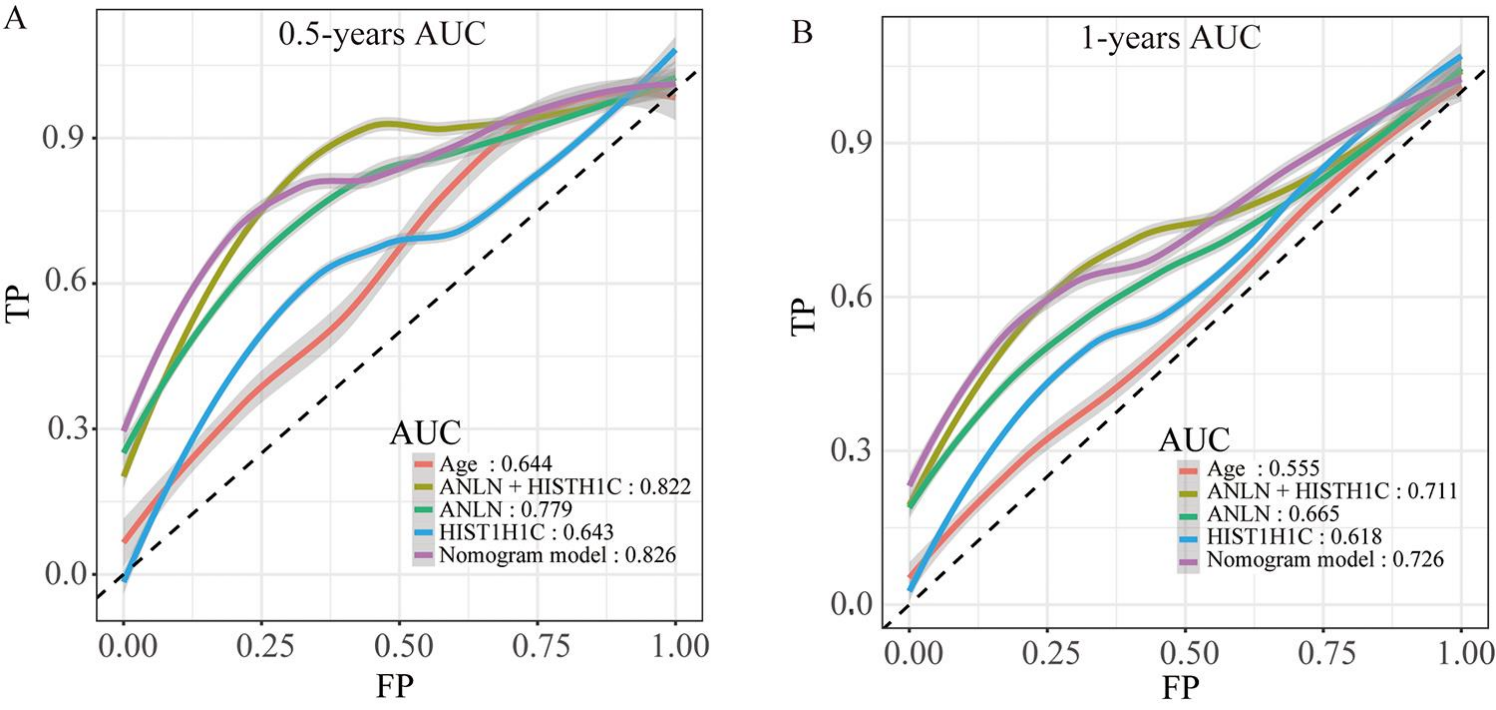
The time-dependent ROC curves of the nomogram and single variables in the model predicting the overall survival after surgery at 0.5 years (**A**) and 1 year (**B**).

To make the prognostic model clinical applicable, a nomogram was applied to predict the probability of the 0.5-, 1-, and 1.5-year OS in the TCGA cohort. The predictive factors in the nomogram include age, margin condition and prognostic model. The C-index of the nomogram model was 0.664, which was higher than the others variables alone (Figure 7A and Supplementary Table 13). Moreover, calibration plots were also used to visualize the performance of the nomograms, with more overlap with the gray-line representing better performance (Figure 7B–7D). The AUCs of the different models were also calculated; the nomogram’s AUC was 0.826 at 0.5 years and 0.726 at 1 year, which was the largest compared to the other models (Figure 8). These findings demonstrated that the nomogram built with the combined model is the best nomogram to predict survival for patients with PC, when compared with nomograms built with a single prognostic factor, and demonstrated significance for facilitating patient counseling, decision-making and follow-up arranging.

## DISCUSSION

Pancreatic cancer is one of the deadliest malignant tumors worldwide, with a gradually increasing morbidity each year. Despite the availability of improving surgical techniques, progress in PC treatment still remains slow, as the 5-year overall survival rate has only improved to 9% in the period 2006 to 2012 in the US [4, 14, 15]. The reasons for this phenomenon could be various, though one specific reason might be critical, namely, the lack of a risk assessment system, which makes it difficult to make therapy decisions for an individual patient with PC.

As is known to all, treatment for pancreatic cancer has experienced some changes from curative resection alone to surgical treatment with adjuvant chemotherapy, which has provided patients with many kinds of therapeutic regimens. In addition, owing to the enormous and high-quality clinical trials focused on adjuvant chemotherapy regimens after surgery, combination chemotherapy has been found to lead to longer survival in select PC patients [16]. However, the greater concomitant toxicity of combination chemotherapy has aroused a new debate: should combination chemotherapy be the first-line chemotherapy for all PC patients, especially when the patients are of a low recurrence risk? Moreover, the course of adjuvant chemotherapy and the frequency of reexamination after discharge also remain controversial [17, 18]. It would be much better if there were an accurate and clinical applicable criterion to assess the possibility of recurrence in PC patients. It is important to note that resectability status alone is not a reliable prognostic factor in PC; even in potential curative resection patients, median survival outcomes were similar to nonradical resection patients [19]. Except for anatomical considerations, CA 19-9 levels are the most commonly used antigen in the clinic to assess the resectability and prognosis of PC patients [20]. However, CA 19-9 is also elevated in other benign conditions and multiple cancer types with limited sensitivity [21]. Several studies have recently characterized whole-genome changes occurring in PC by analyzing the mutational landscape of these deadly diseases [21]. Owing to these findings, the discovery of gene signatures to assess PC patient prognosis is of practicable and great value.

Recently, using the overlapping analysis, Yan et al. [22] identified four survival-related genes (*LYRM1*, *KNTC1*, *IGF2BP2* and *CDC6*) in four public PC datasets. And the predictive nomogram based these four survival-related genes shows robust performance in predicting PC prognosis. However, the disturbances arising from outliers do not fit well in their framework. Therefore, more reliable predictive model needs to be built and optimized. In our study, we selected genes that were significantly highly/lowly expressed in all three datasets. Among the 35 survival-related genes, aside from the two genes that have not been reported to have a relationship with tumor progression or prognosis in any cancer types, 16 (45.7%) genes have been reported to be related to tumor progression and prognosis. Moreover, 11 (31.4%) genes have been reported to be related with the occurrence and outcome of PC, which demonstrated the correctness and repeatability of our data mining methods and results. To further minimize the scope of the survival-related genes, we applied multivariate Cox regression complex with a Lasso regression. To reduce the overfitting and avoid disturbances arising from outliers, we also used a 10-fold cross validation and performed the whole analytical process 3000 times. We selected the most frequent genes (*ANLN* and *HIST1H1C*) to construct the predicting model, which is different from the model conducted by Yan’s group [22]. Then, we proved that the prognostic signature performed well for the discrimination of the high and low risk groups using both the TCGA PC datasets and GEO datasets. At the same time, the AUCs of the two-gene biomarker prognostic model were plotted, which demonstrated the acceptable predictive value of the two genes. Consistent with previous studies, the AJCC staging system allowed the prediction of clinical outcomes for PC patients. In spite of the anatomical extent of the cancer, which was assessed using a staging system, biological heterogeneity might play a critical role in pancreatic carcinoma. Compared with a single factor, our nomogram is more powerful for prediction and may became a reference item in the clinic in the future.

*ANLN*, an actin-binding protein, is essential for assembly of the cleavage furrow during the late stages of mitosis and acts as the central organizer at the cytokinetic machinery hub [23]. Owing to its critical role, studies have observed overexpressed *ANLN* in several types of cancers [24–26], and it has also been proven to be correlated with poor prognoses in breast cancer, lung cancer and hepatocellular carcinoma [27–29]. However, the role of *ANLN* in PC remains unclear. To the best of our knowledge, our study was the first to validate that high-expressed *ANLN* is associated with low overall survival by means of bioinformatic analysis. The pathogenesis of *ANLN* in tumor progression might act as a cell cycle regulator, enabling the promotion of tumor growth by decreasing apoptosis and DNA damage, though the detailed mechanisms require further investigation [30]. *HIST1H1C*, another PC molecular marker, is a basic nuclear protein responsible for interaction with the linker DNA between nucleosomes and functions to compact chromatin into higher order structures. Until now, however, only one study has reported *HIST1H1C* as a hub gene among the DEGs in nonfunctional pituitary adenomas, and one study illustrated that *HIST1H1C* is involved with tumor growth in pancreatic cancer [31]. There are few studies focused on *HIST1H1C*, which makes the significance of this gene in PC uncertain and worthy of future investigations.

Nevertheless, there are some limitations of our study. First, considering the poor prognosis of pancreatic carcinomas, there were not enough patients with an OS over 3 years, meaning that this approach could be inaccurate if we are going to predict the more long-term outcomes from patients with PC. Second, due to the lack of particular clinical data in GSE28735 and GSE62452, we were unable to perform external validation of our nomogram in those GES databases, which means the nomogram should be further validated using multicenter clinical trials and prospective studies.

In conclusion, two genes have been identified as having a prognostic significance in pancreatic carcinoma using a relatively rigid regression model method. For the first time, we report that *ANLN* and *HIST1H1C* are related to the clinical outcome of PC patients. We also constructed a nomogram comprising the prognostic models to assist clinicians treating patients with PC in a personalized way.

## MATERIALS AND METHODS

### Patients and samples

The mRNA expression and corresponding clinical data of PC patients were obtained from the TCGA dataset that contained 178 PC tissues and 4 adjacent noncancerous pancreatic tissues, which is in accordance with the report from Lu’s group [32]. This TCGA dataset was downloaded from Genomic Data Commons Data Portal (https://portal.gdc.cancer.gov/) at January 2019. The processed mRNA expression data of patients with pancreatic ductal adenocarcinoma were downloaded from two GEO datasets (GSE28735 and GSE62452) [33, 34] that contained the microarray gene-expression profiles of 69 pancreatic tumors and 61 adjacent nontumor tissues as well as the microarray gene-expression profiles of 45 matching pairs of pancreatic tumors and adjacent nontumor tissues from 45 separate patients. We screened potential GEO datasets according to the following inclusion criteria: 1) PC samples and nonmalignant adjacent tissues, 2) studies with more than 45 PC samples or adjacent noncancerous tissues, 2) expression profiling by array, and 3) all samples from Homo sapiens. As afore mentioned [10], the datasets with samples from other organisms or cell lines, those that performed the expression profiling by high-throughput sequencing, those with non-coding RNA profiling by high-throughput sequencing, those with genome variation profiling by genome tiling array, and those with methylation profiling by array or single nucleotide polymorphism genotyping by array were excluded.

### RNA-seq data quantification and analysis

Initially, the raw PC mRNA expression profile counts were downloaded from the TCGA database (https://portal.gdc.cancer.gov/), GSE28735 and GSE62452 (https://www.ncbi.nlm.nih.gov/gds). Second, we calculated the DEGs from the TCGA data by means of the limma package and from the GEO data by means of the “edgeR” package. The DEGs from the datasets with a P<0.01 were selected and a Venn diagram was plotted. Only the DEGs in all three datasets were considered for subsequent analysis.

### Functional enrichment analysis

The Gene Ontology (GO) and Kyoto Encyclopedia of Genes and Genomes (KEGG) pathway enrichment analysis of the common DEGs in the three datasets were performed using the R package “clusterProfiler” for Annotation and Visualization.

### Constructing the gene-related prognostic model

We initially employed a univariate COX regression analysis of the TCGA datasets to investigate the correlation between patient OS and the expression levels of each gene, which was considered to be significant when the P<0.05. We then used the R package “glmnet” to perform the Lasso-penalized Cox regression along with a 10-fold cross-validation to further screen significant genes with prognosis [35, 36]. During the Lasso-penalized Cox regression selection, we randomly subsampled 70% of TCGA patients with 3000 replacements and selected the genes with repeat occurrence frequencies greater than 2500 times. We adopted the largest lambda value such that the error was within one standard error of the minimum, which was called “1-se” lambda [37]. Subsequently, a multivariate Cox regression analysis with a stepwise method was performed to assess the contribution of a gene as an independent prognosis factor for patient survival and to further select the best model. The robustness of all the above-mentioned models was validated by bootstrap procedures [38]. Finally, the risk score (RS) of the mRNA signature was calculated according to the following formula: $\text{RiskSore}=\sum_{i=1}^{n}\left(coef_{i}\times Expr_{i}\right),$ where Expr was the mRNA expression and coef is the mRNA Lasso coefficient. The optimal cut-off value of RS was determined by X-tile plots version 3.6.1 (Yale University School of Medicine, New Haven, CT, USA) [39]. Based on the cut-off value of RS, patients were classified into the low-risk group and high-risk group, and the K-M curves for the different groups were plotted. Time-dependent receiver operating characteristic (ROC) curve analysis was performed to evaluate the predictive value of the prediction model. Furthermore, the prognostic gene signature was also validated using the GEO dataset (GSE28735).

### Independence of the prognostic gene signature from other clinical characteristics

To prove the independent predictive power of the prognostic gene signature when other clinical variables (such as age, sex, histologic grade, AJCC stage, tumor size and tumor residual) were present in patients with PC, univariate and multivariate Cox regression analyses were performed with the clinical characteristics and gene prognostic model set as independent variables and the OS set as the dependent variable. The robustness of all the above-mentioned models was also validated by bootstrap procedures [38]. All reported P values were two-sided. The hazard ratio (HR) and 95% confidence intervals (CI) were calculated.

### Building and validating a predictive nomogram

To improve the generalization ability and applicability of the model, we combined clinical variables with prognostic power with the gene signature model, and we applied the nomogram for the prediction of PC patient prognosis. In this study, the combined model based on all independent prognostic factors selected by the multivariable Cox regression analysis was used to construct a nomogram to assess the probability of the 0.5-, 1-, and 1.5-year OS for patients with PC. Subsequently, the C-index was calculated using R with the “survival” package. Then, to prove that the combined model has an advantage over other single variables, time-dependent ROC curves were obtained using the R package “pROC” [40].

### Statistical analyses

The levels of *ANLN* and *HIST1H1C* that were differentially expressed between cancerous and adjacent noncancerous pancreatic tissues were estimated using Student’s t-test with SPSS 12.0 software. In addition, the significantly annotated KEGG and GO were calculated in clusterProfiler, and false discovery rate was analyzed using Benjamini correction. Significance of survival analysis was performed by K-M curve with log-rank test. A P value less than 0.05 is considered statistically significant.

## Supplementary Material

Supplementary Figure 1

Supplementary Table 1

Supplementary Table 2

Supplementary Table 3

Supplementary Table 4

Supplementary Table 5

Supplementary Table 6

Supplementary Table 7

Supplementary Table 8

Supplementary Table 9

Supplementary Table 10

Supplementary Tables 11, 12 and 13
