# Supplementary material for: A two-gene-based prognostic signature for pancreatic cancer
Source: Aging (Albany NY). 2020 Sep 23;12(18):18322–42. doi: 10.18632/aging.103698 (PMC7585105; doi:10.18632/aging.103698)
Supplement: Supplementary Figure 1 [file aging-12-103698-s012..pdf]

SUPPLEMENTARY FIGURE

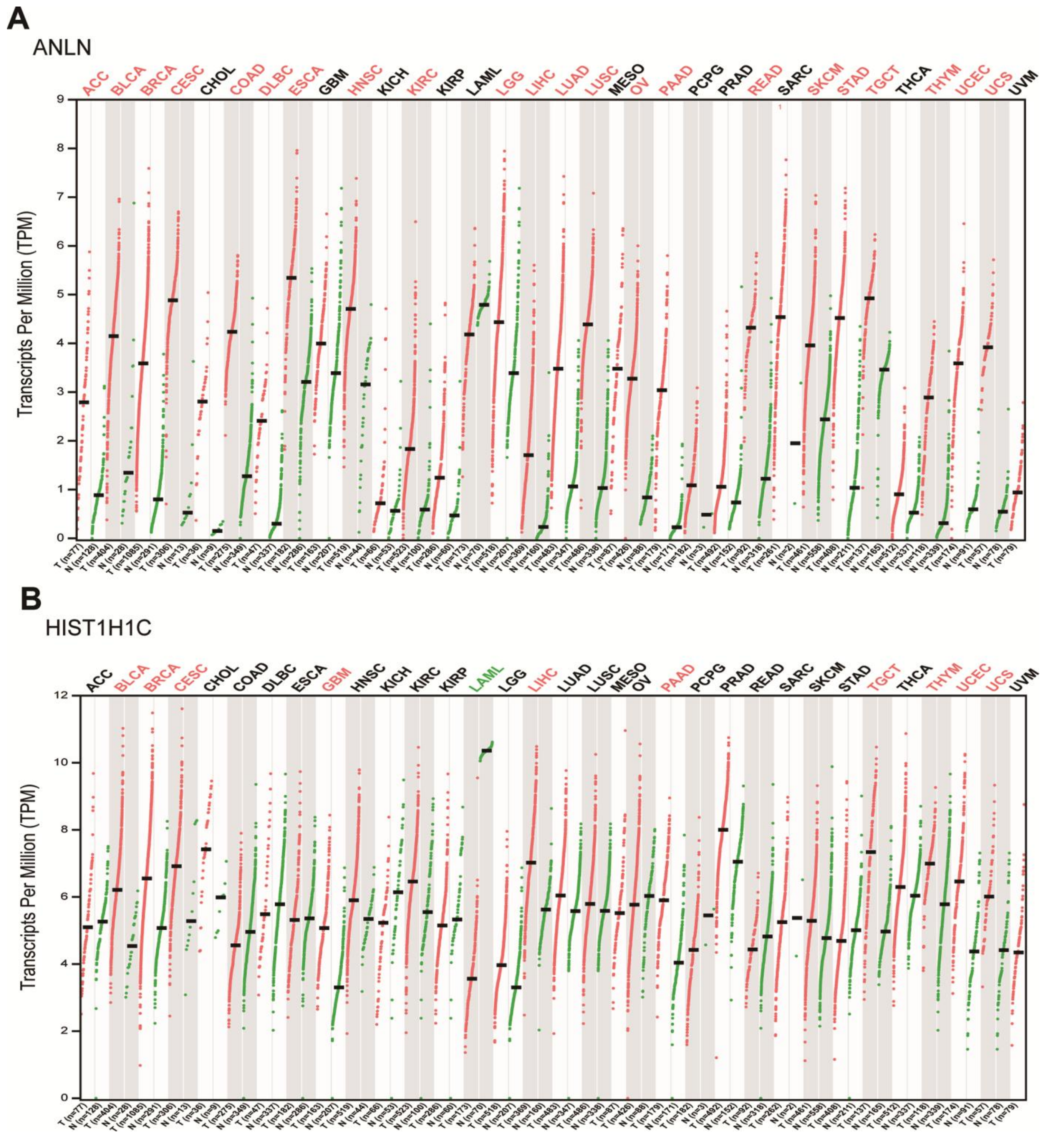

Supplementary Figure 1. The pancancer analysis of *ANLN* and *HIST1H1C* expression across multiple cancer types in GEPIA 2.0 database. Red tumor abbreviations indicated significant upregulation of *ANLN* and *HIST1H1C* in cancers, while green tumor abbreviations indicated significant downregulation of *ANLN* and *HIST1H1C.*
