# Supplementary material for: A two-gene-based prognostic signature for pancreatic cancer
Source: Aging (Albany NY). 2020 Sep 23;12(18):18322–42. doi: 10.18632/aging.103698 (PMC7585105; doi:10.18632/aging.103698)
Supplement: Supplementary Table 5 [file aging-12-103698-s011..pdf]

## SUPPLEMENTARY TABLE

**Supplementary Table 5. 3000 times lasso cox regression genes.**

| | Gene | Times |
|---|---|---|
| 1 | ECT2 | 316 |
| 2 | ANLN | 2640 |
| 3 | HIST1H2BD | 0 |
| 4 | NEK2 | 0 |
| 5 | HIST1H2AC | 326 |
| 6 | CD36 | 1692 |
| 7 | HIST1H2BJ | 0 |
| 8 | ZMAT1 | 0 |
| 9 | CHEK1 | 0 |
| 10 | KIF4A | 0 |
| 11 | CDC6 | 0 |
| 12 | BCL11A | 0 |
| 13 | HIST1H2BC | 0 |
| 14 | CLK4 | 0 |
| 15 | HIST1H1C | 2543 |
| 16 | HIST1H2BK | 0 |
| 17 | PLCG2 | 312 |
| 18 | MAP3K14 | 1 |
| 19 | GSDMC | 0 |
| 20 | SCML4 | 0 |
| 21 | ZBTB32 | 0 |
| 22 | HRASLS2 | 0 |
| 23 | HIST1H4I | 0 |
| 24 | GDPD5 | 30 |
| 25 | GYPC | 0 |
| 26 | RNF166 | 0 |
| 27 | CCM2L | 0 |
| 28 | LIMD2 | 948 |
| 29 | OXER1 | 0 |
| 30 | IL16 | 1673 |
| 31 | PSTPIP1 | 0 |
| 32 | AGER | 0 |
| 33 | VENTX | 0 |
| 34 | GH1 | 0 |
| 35 | BTNL9 | 0 |
