# Supplementary material for: A two-gene-based prognostic signature for pancreatic cancer
Source: Aging (Albany NY). 2020 Sep 23;12(18):18322–42. doi: 10.18632/aging.103698 (PMC7585105; doi:10.18632/aging.103698)
Supplement: Supplementary Table 9 [file aging-12-103698-s007..docx]

SUPPLEMENTARY TABLE

Supplementary Table 9. The expression of ANLN and HIST1H1C in PC cells
from Cancer Cell Line Encyclopedia (CCLE).

| **Gene** | **ANLN** | **HIST1H1C** |
| --- | --- | --- |
| SNU410_PANCREAS | 7.595567 | 5.063576 |
| PANC0403_PANCREAS | 7.45979 | 3.238598 |
| PANC1_PANCREAS | 7.032564 | 4.064839 |
| PK45H_PANCREAS | 6.952955 | 5.389367 |
| SW1990_PANCREAS | 6.893646 | 4.444045 |
| PATU8902_PANCREAS | 6.839072 | 3.806673 |
| SNU213_PANCREAS | 6.79091 | 3.951536 |
| KP2_PANCREAS | 6.65843 | 5.718855 |
| HS766T_PANCREAS | 6.294668 | 6.366739 |
| PANC0203_PANCREAS | 6.291476 | 4.468557 |
| PANC1005_PANCREAS | 6.249681 | 5.16116 |
| PATU8988T_PANCREAS | 6.243406 | 4.120279 |
| T3M4_PANCREAS | 6.232112 | 6.643391 |
| KP3_PANCREAS | 6.17958 | 4.369459 |
| PK1_PANCREAS | 6.102597 | 3.550379 |
| PANC0327_PANCREAS | 6.077964 | 6.297954 |
| YAPC_PANCREAS | 6.05971 | 3.701167 |
| HUPT3_PANCREAS | 6.056466 | 6.20422 |
| KP4_PANCREAS | 5.910993 | 6.217787 |
| PANC0504_PANCREAS | 5.89602 | 4.113109 |
| SUIT2_PANCREAS | 5.895513 | 3.811658 |
| TCCPAN2_PANCREAS | 5.875302 | 6.281926 |
| PANC0213_PANCREAS | 5.815443 | 4.933527 |
| CAPAN2_PANCREAS | 5.763245 | 3.244718 |
| MIAPACA2_PANCREAS | 5.712785 | 5.638969 |
| PANC0813_PANCREAS | 5.665741 | 4.26619 |
| SNU324_PANCREAS | 5.623243 | 4.435857 |
| PK59_PANCREAS | 5.584925 | 4.309652 |
| CAPAN1_PANCREAS | 5.458695 | 6.691079 |
| BXPC3_PANCREAS | 5.431424 | 5.106647 |
| CFPAC1_PANCREAS | 5.410521 | 6.612041 |
| HPAFII_PANCREAS | 5.405624 | 4.803013 |
| L33_PANCREAS | 5.322749 | 4.259711 |
| HPAC_PANCREAS | 5.19894 | 3.818123 |
| HUPT4_PANCREAS | 5.196609 | 3.970606 |
| DANG_PANCREAS | 5.165471 | 3.057551 |
| PATU8988S_PANCREAS | 5.028114 | 4.90915 |
| PSN1_PANCREAS | 4.380796 | 3.110652 |
| ASPC1_PANCREAS | 4.184791 | 3.775559 |
| SU8686_PANCREAS | 3.933033 | 2.336863 |
| QGP1_PANCREAS | 3.925441 | 4.861757 |
