# Supplementary material for: A two-gene-based prognostic signature for pancreatic cancer
Source: Aging (Albany NY). 2020 Sep 23;12(18):18322–42. doi: 10.18632/aging.103698 (PMC7585105; doi:10.18632/aging.103698)
Supplement: Supplementary Tables 11, 12 and 13 [file aging-12-103698-s010..pdf]

SUPPLEMENTARY TABLES

Supplementary Table 11. The two-gene expression risk stratification in GSE28735.

	ID	HIST1H1C	ANLN	Surtime	Surstat	Riskscore	Groupby
1	GSM711904	4.59351	3.478818	4.25	1	0.401283	Low risk
2	GSM711906	5.383048	6.50617	0.58	1	1.851105	High risk
3	GSM711908	4.642691	6.541408	0.25	1	3.62942	High risk
4	GSM711910	3.707255	2.865696	3.5	1	0.555705	Low risk
5	GSM711914	4.629382	5.674897	3	1	1.945416	High risk
6	GSM711916	4.828843	4.864498	0.17	1	0.902076	Low risk
7	GSM711922	4.808846	4.835817	1.58	1	0.89889	Low risk
8	GSM711924	4.515929	5.470503	1.08	1	1.849385	High risk
9	GSM711926	4.718713	4.651367	1.33	1	0.849599	Low risk
10	GSM711928	4.044202	4.589405	3.42	1	1.464419	Low risk
11	GSM711930	4.380305	5.038249	0.25	1	1.516759	High risk
12	GSM711932	4.133895	6.320266	1	1	4.815825	High risk
13	GSM711934	4.482482	4.605157	2.08	1	1.009769	Low risk
14	GSM711936	4.709863	2.9729	3.17	0	0.250142	Low risk
15	GSM711938	4.472675	2.621358	0.08	0	0.237873	Low risk
16	GSM711940	5.041278	5.580661	1.08	1	1.266404	Low risk
17	GSM711942	4.77368	5.394913	1.92	1	1.396585	Low risk
18	GSM711944	4.523715	5.181269	0.92	1	1.485881	Low risk
19	GSM711946	3.95196	3.259936	2.42	1	0.598995	Low risk
20	GSM711948	4.34825	3.772391	2.33	1	0.616691	Low risk
21	GSM711950	4.441237	3.769534	2.33	0	0.567338	Low risk
22	GSM711952	5.284748	5.607847	1.17	1	1.044138	Low risk
23	GSM711954	4.415777	5.321105	0.58	1	1.809254	High risk
24	GSM711956	5.069863	4.410287	2.33	0	0.523712	Low risk
25	GSM711958	5.338916	5.728907	2	0	1.088248	Low risk
26	GSM711960	4.75965	4.458094	2	0	0.71143	Low risk
27	GSM711962	4.692492	6.405433	0.67	1	3.145077	High risk
28	GSM711964	4.87009	5.140327	1.83	0	1.065114	Low risk
29	GSM711966	5.500636	5.748288	1.75	0	0.958246	Low risk
30	GSM711968	4.71547	5.310038	1.75	0	1.380871	Low risk
31	GSM711970	5.056518	4.657617	0.75	1	0.635193	Low risk
32	GSM711972	5.393925	6.276303	1.42	0	1.549226	High risk
33	GSM711974	4.565349	5.451879	0.5	1	1.747143	High risk
34	GSM711976	4.734871	4.955239	1.33	0	1.046703	Low risk
35	GSM711978	4.637025	4.249571	0.42	1	0.679697	Low risk
36	GSM711980	4.19272	4.349029	0.92	0	1.07827	Low risk
37	GSM711982	4.914539	6.970197	0.33	1	3.918385	High risk
38	GSM711984	4.047127	2.808281	0.83	1	0.395805	Low risk
39	GSM711986	5.846707	5.333412	0.83	0	0.522338	Low risk
40	GSM711988	4.780226	4.680945	1.25	1	0.82275	Low risk
41	GSM711990	4.518276	3.429367	0.42	1	0.413314	Low risk
42	GSM711992	4.661236	4.14586	1.08	1	0.616738	Low risk

Supplementary Table 12. The two-gene expression risk stratification in GSE62452.

	Sample	Fultime	Fustate	Type	Express_ANLN	Express_HIST1HIC	Riskscore	Groupby
1	GSM1527105	4.258333	1	Tumor	3.28581	4.15587	0.69245	Low risk
2	GSM1527107	0.575	1	Tumor	6.37726	4.98483	3.641451	High risk
3	GSM1527109	0.225	1	Tumor	6.27981	4.12032	1.334185	High risk
4	GSM1527111	3.466667	1	Tumor	2.72845	3.3195	0.235374	Low risk
5	GSM1527115	2.991667	1	Tumor	5.47523	4.14786	1.141673	High risk
6	GSM1527117	0.2	1	Tumor	4.65815	4.3633	1.20571	High risk
7	GSM1527123	1.625	1	Tumor	4.63507	4.33638	1.163162	High risk
8	GSM1527125	1.05	1	Tumor	5.27076	4.1233	1.058728	High risk
9	GSM1527127	1.333333	1	Tumor	4.42231	4.23132	0.982557	High risk
10	GSM1527129	3.408333	1	Tumor	4.39364	3.68734	0.526347	Low risk
11	GSM1527131	0.233333	1	Tumor	4.76962	3.9566	0.779813	Low risk
12	GSM1527133	0.966667	1	Tumor	6.14909	3.71616	0.818008	Low risk
13	GSM1527135	2.058333	1	Tumor	4.45058	4.10903	0.860839	Low risk
14	GSM1527137	3.333333	0	Tumor	2.82307	4.17296	0.633992	Low risk
15	GSM1527139	0.1	1	Tumor	2.48363	3.94183	0.450665	Low risk
16	GSM1527141	1.1	1	Tumor	5.38821	4.62335	1.919456	High risk
17	GSM1527143	1.933333	1	Tumor	5.20154	4.40832	1.439838	High risk
18	GSM1527145	0.9	1	Tumor	5.02687	4.06697	0.93839	Low risk
19	GSM1527147	2.416667	1	Tumor	3.0661	3.52321	0.32084	Low risk
20	GSM1527149	2.308333	1	Tumor	3.55992	3.9235	0.566902	Low risk
21	GSM1527151	2.3	0	Tumor	3.56466	3.98591	0.609194	Low risk
22	GSM1527154	1.15	1	Normal	2.30865	3.24949	0.197173	Low risk
23	GSM1527155	0.566667	1	Tumor	5.07569	4.01034	0.890008	Low risk
24	GSM1527157	2.35	0	Tumor	4.21571	4.71055	1.613475	High risk
25	GSM1527159	0.816667	1	Tumor	5.63999	4.9959	3.106545	High risk
26	GSM1527161	1.966667	0	Tumor	4.26289	4.30718	1.03195	High risk
27	GSM1527163	0.641667	1	Tumor	6.21958	4.25898	1.539909	High risk
28	GSM1527165	1.816667	0	Tumor	4.95109	4.51506	1.533367	High risk
29	GSM1527167	1.766667	0	Tumor	5.56318	5.1566	3.662255	High risk
30	GSM1527169	1.758333	0	Tumor	5.08181	4.21906	1.129573	High risk
31	GSM1527171	0.741667	1	Tumor	4.47359	4.67485	1.645144	High risk
32	GSM1527173	1.441667	0	Tumor	6.17811	5.04493	3.722144	High risk
33	GSM1527175	0.533333	1	Tumor	5.21309	4.07266	0.986266	High risk
34	GSM1527177	1.366667	0	Tumor	4.75762	4.28066	1.123428	High risk
35	GSM1527179	0.383333	1	Tumor	4.04649	4.23222	0.901257	Low risk
36	GSM1527181	0.883333	0	Tumor	4.1425	3.71913	0.514734	Low risk
37	GSM1527183	0.35	1	Tumor	6.79223	4.4891	2.284431	High risk
38	GSM1527185	0.858333	1	Tumor	2.80611	3.64402	0.346414	Low risk
39	GSM1527187	0.808333	0	Tumor	5.15503	5.44705	4.631561	High risk
40	GSM1527189	1.241667	1	Tumor	4.50642	4.38521	1.193225	High risk
41	GSM1527191	0.375	1	Tumor	3.26562	4.09829	0.645597	Low risk
42	GSM1527193	1.075	1	Tumor	3.95943	4.3022	0.956221	Low risk
43	GSM1527196	0.791667	1	Tumor	4.54332	3.84922	0.654933	Low risk
44	GSM1527198	0.525	1	Tumor	5.90911	4.32916	1.551464	High risk
45	GSM1527199	0.075	1	Tumor	4.35018	4.08732	0.820502	Low risk
46	GSM1527200	0.491667	1	Tumor	3.4362	3.42219	0.311788	Low risk
47	GSM1527202	0.816667	1	Tumor	4.83738	4.44265	1.375509	High risk
48	GSM1527204	0.441667	1	Tumor	6.22746	4.1379	1.344611	High risk

49	GSM1527205	1.791667	1	Tumor	3.64831	4.32354	0.911297	Low risk
50	GSM1527207	1.183333	1	Tumor	6.01381	4.34821	1.624452	High risk
51	GSM1527209	2.666667	1	Tumor	5.50742	4.31662	1.393137	High risk
52	GSM1527210	1.908333	1	Tumor	5.93411	5.57191	6.39679	High risk
53	GSM1527212	3.825	1	Tumor	3.47327	3.88402	0.531243	Low risk
54	GSM1527213	1.825	1	Tumor	5.24938	3.28717	0.407756	Low risk
55	GSM1527215	3.5	0	Tumor	3.08215	3.39736	0.279167	Low risk
56	GSM1527216	3.191667	0	Tumor	3.19303	3.81927	0.462455	Low risk
57	GSM1527218	1.141667	1	Tumor	7.66299	4.93645	4.647977	High risk
58	GSM1527219	0.908333	1	Tumor	5.13991	4.24769	1.18276	High risk
59	GSM1527220	1.775	1	Tumor	6.3008	4.59253	2.291621	High risk
60	GSM1527223	0.775	1	Tumor	3.63132	3.69955	0.447	Low risk
61	GSM1527225	1.658333	1	Tumor	3.98875	4.95504	2.020132	High risk
62	GSM1527227	5.9	0	Tumor	3.59565	3.98568	0.61344	Low risk
63	GSM1527228	5.666667	0	Tumor	3.56692	4.14986	0.734199	Low risk
64	GSM1527230	5.641667	0	Tumor	3.65724	4.28682	0.875908	Low risk
65	GSM1527232	4.141667	1	Tumor	3.11183	4.27017	0.75713	Low risk
66	GSM1527234	0.266667	1	Tumor	3.60256	4.10509	0.703624	Low risk

Supplementary Table 13. The C-index of nomogram model.

**Univariate analysis**					
Age(36-84)	1.00	1.05	0.018	1.03	1.03(1.00,1.05)
Sex(Male/Female)	0.56	1.42	0.628	0.99	0.99(0.56,1.42)
Grade(G1-2/G3-4)	0.60	1.59	0.929	1.10	1.10(0.60,1.59)
Stage(III-IV/I-II)	0.17	2.07	0.417	1.12	1.12(0.17,2.07)
Size(1.8-12)	0.92	1.34	0.259	1.13	1.13(0.92,1.34)
Residual(R0/non-R0)	0.39	1.01	0.054	0.70	0.70(0.39,1.01)
Prognostic model()	1.43	2.16	0.000	1.80	1.80(1.43,2.16)
**Multivariate analysis**		0.000			
Age	1.00	1.05	0.021	1.03	1.03(1.00,1.05)
Residual	0.40	1.03	0.067	0.72	0.72(0.40,1.03)
Riskscore	1.48	2.21	0.000	1.85	1.85(1.48,2.21)

**Items**	**C-index**
Age	0.55
Residual	0.577
riskscore	0.647
nomogram model	0.664
ANLN	0.633
HIST1H1C	0.553
